# Prevalent Cardiovascular Disease and Atrial Fibrillation in Relation to Cerebral Small Vessel Disease Burden

**DOI:** 10.3390/brainsci15080813

**Published:** 2025-07-29

**Authors:** Oluchi Ekenze, Adlin Pinheiro, Alexa S. Beiser, Vasileios-Arsenios Lioutas, Hugo J. Aparicio, Emelia J. Benjamin, Ramachandran S. Vasan, Charles DeCarli, Sudha Seshadri, Serkalem Demissie, Jose R. Romero

**Affiliations:** 1 NHLBI’s Framingham Heart Study, Framingham, MA 01702, USA; adlinp@bu.edu (A.P.); alexab@bu.edu (A.S.B.); vlioutas@bidmc.harvard.edu (V.-A.L.); hugoa@bu.edu (H.J.A.); emelia@bu.edu (E.J.B.); seshadri@uthscsa.edu (S.S.); demissie@bu.edu (S.D.); joromero@bu.edu (J.R.R.); 2 Department of Neurology, Boston University Chobanian & Avedisian School of Medicine, Boston, MA 02118, USA; 3 Department of Biostatistics, Boston University School of Public Health, Boston, MA 02118, USA; 4 Department of Neurology, Beth Israel Deaconess Medical Center, Harvard Medical School, Boston, MA 02215, USA; 5 Section of Cardiovascular Medicine, Boston Medical Center, Boston University Chobanian & Avedisian School of Medicine, Department of Epidemiology, Boston University School of Public Health, Boston, MA 02118, USA; 6 School of Public Health, University of Texas at San Antonio, San Antonio, TX 78229, USA; vasan@uthscsa.edu; 7 Department of Neurology, University of California at Davis, Davis, CA 95616, USA; cdecarli@health.ucdavis.edu; 8 The Glenn Biggs Institute for Alzheimer’s and Neurodegenerative Diseases, University of Texas Health Sciences Center, San Antonio, TX 78229, USA

**Keywords:** prevalent cardiovascular disease, atrial fibrillation, cerebral small vessel disease

## Abstract

Background/Objectives: Cardiovascular disease (CVD) contributes to stroke and dementia. Individuals with CVD have high risk for adverse cognitive outcomes and stroke, possibly due to shared risk factors between CVD, stroke, and dementia, which may be attributed to cerebral small vessel disease (CSVD). We aim to determine the association between prevalent CVD and atrial fibrillation (AF) with CSVD. Methods: Composite of CVD [coronary heart disease, heart failure (HF)], its individual components, and AF were assessed. Multi-marker CSVD score was used to reflect increasing CSVD burden (cerebral microbleeds (CMBs), high-burden perivascular spaces, extensive white matter hyperintensity, cortical superficial siderosis, or covert brain infarcts were assigned 1 point each, with a range of 0–5). We related prevalent CVD, its individual components, and AF to multi-marker CSVD score and individual CSVD markers using logistic regression analyses adjusted for age, sex, FHS cohort, time between MRI and clinic exam (model-1), and vascular risk factors (model-2). Results: In 3413 participants (mean age: 59 ± 14 years, 53.4% women), 11% had prevalent CVD or AF, 8% had prevalent CVD, and 4% had prevalent AF. One CSVD marker was seen in 23% participants, and 9% had ≥ 2 markers. In multivariable-adjusted analyses, composite prevalent CVD and AF was associated with the presence of one CSVD marker (OR: 1.38, 95% confidence interval [CI]: 1.05–1.84). The association with ≥2 CSVD markers was not significant. Only CMBs were associated with components of CVD and AF, with the highest odds of association with HF. Conclusions: Prevalent CVD (including AF) is associated with the presence of CSVD, with all components associated with CMBs.

## 1. Introduction

Cardiovascular disease (CVD) is the largest contributor to global mortality and reduced quality of life with increased burden in the elderly [1,2]. About 128 (48.6%) million Americans over 20 years old have CVD [1]. Individuals with established CVD are at the highest risk for adverse cognitive outcomes and recurrent stroke due to shared risk factors between atherosclerotic CVD, stroke, and dementia [3]. For example, midlife hypertension and type 2 diabetes are risk factors for stroke, cardiovascular disease, and dementia [3,4]. Thus, those with prevalent CVD may have a higher risk of dementia and stroke due to ongoing vascular brain injury from exposure to vascular risk factors. Assessment of subclinical cerebrovascular disease may help in prevention of adverse events in these patients. Atrial fibrillation (AF) is associated with stroke, dementia, silent brain infarcts, and white matter injury [5,6,7]. However, its association with other cerebral small vessel disease remains underexplored.

Cerebral small vessel disease (CSVD), a major subtype of vascular brain injury strongly linked to a higher risk of stroke and dementia, can be assessed in subclinical stages, years to decades before clinical stroke and dementia occur [8,9]. Although CSVD is strongly related to vascular risk factors such as hypertension, its relation to stroke and dementia risk remains significant even after adjustment for such risk factors measured on cross-sectional assessments, suggesting that CSVD may assess better an individual’s risk compared to vascular risk factors [10].

CSVD is characterized using magnetic resonance imaging (MRI) by various individual markers: white matter hyperintensities (WMHs), lacunes, cerebral microbleeds (CMBs), brain atrophy, enlarged perivascular spaces (PVSs), cortical superficial siderosis, and microinfarcts [11]. Recently, CSVD scores that incorporate individual markers of CSVD have been used to better reflect the overall burden of CSVD with an increased CSVD score linked to a higher risk of stroke and dementia compared to individual markers [12].

We aimed to study the relation between prevalent CVD and AF with multi-marker CSVD scores, to test the hypothesis that individuals with prevalent CVD or AF have a higher multi-marker CSVD score. Findings from this study may help emphasize the need for enhanced surveillance of persons with established CVD and AF who might be at an increased risk for ongoing subclinical vascular injury.

## 2. Materials and Methods

### 2.1. Sample

The recruitment of participants in the Framingham Heart Study (FHS) and MRI acquisition have been previously described [13]. Briefly, the FHS started in 1948 with the recruitment of the original cohort. In 1971, the offspring cohort (offspring of original cohort) and their spouses were recruited. The Omni 1 (more ethnic and racially diverse) cohort was recruited in 1994. Gen 3 (grandchildren of the original cohort) was recruited in 2002. In 2003, the New Offspring Spouse (NOS) cohort (consisting of spouses of offspring participants who had never been recruited and had at least two biological children that participated in exam 1 of Gen 3) was recruited.

The FHS participants were examined, on average, every 2–8 years (original cohort: 2 years; offspring: 4–8 years; Gen 3 and NOS: 6 years; Omni 1: 4–8 years) [13], with cardiovascular-targeted physical examination, measurement of anthropometric data, and 12-lead electrocardiography (ECG). Since 1999, brain MRI has been performed as part of ancillary studies on brain structures [13].

**Figure 1:**
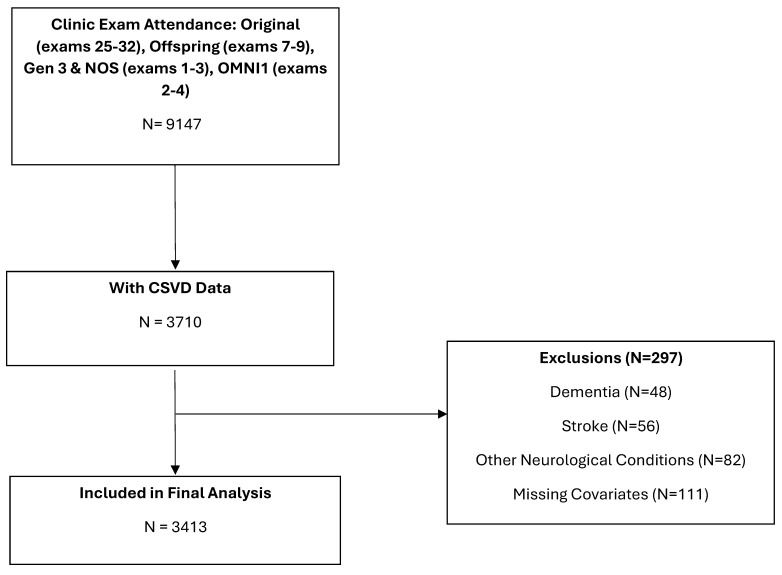
Flow chart of sample selection.

We included original, offspring, Gen 3, NOS, and Omni 1 cohorts who attended at least one clinic exam in which brain MRI was performed during the period of 1999–2019. All participants were invited for brain MRI during their examination cycles and included if they had brain MRI. Brain MRIs were conducted within a one-year period before or up to five years following the clinical examination. Of the 9147 participants who attended the clinic exam (original cohort exam: 25–32; offspring exam: 7–9; Gen 3 and NOS exam: 1–3; Omni 1 exam: 2–4), 3710 had at least one MRI with assessment for all CSVD markers. The final sample consisted of 3413 participants after excluding 297 for history of stroke, dementia, or other neurological conditions. The first available MRI was selected for participants who had more than one MRI with CSVD marker ratings. Participants sample selection is shown in Figure 1.

### 2.2. Exposure

Prevalent CVD is defined as the presence of coronary heart disease (stable angina, coronary insufficiency, and myocardial infarction) or heart failure. These diagnoses were adjudicated according to the established FHS criteria by a panel of three physicians from the endpoints review committee [13]. Atrial fibrillation was diagnosed if at least two FHS cardiologists confirmed rhythm abnormality based on ECG, including external Holter ECGs, telemetry, or other monitoring data.

### 2.3. Brain MRI

Brain MRI acquisition measures and image processing methods have been described in detail [14]. Brain MRI was acquired using a 1T (1999–2005), 1.5T (after 2005) or 3T (after 2010) Magnetom scanner (Siemens Medical, Erlangen, Germany).

### 2.4. Cerebral Small Vessel Disease Markers

Cerebral microbleeds (CMBs) were defined as rounded or ovoid hypointense lesions <10 mm in diameter and surrounded by brain parenchyma over at least half of the circumference on the T2*-GRE-weighted sequence [15]. Inter-rater and intra-rater reliability measures for CMBs in the FHS are excellent (Kappa: 0.78) [10].

Perivascular spaces (PVSs) were rated following the STandards for Reporting Vascular changes on NEuroimaging (STRIVE 1) criteria [11] as high signal intensities similar to cerebrospinal fluid on T2 sequences seen commonly in the basal ganglia (BG), centrum semiovale (CSO), and midbrain [16]. The PVS burden was categorized using an ordinal scale into grades I–IV in BG and CSO based on PVS counts: grade I (1–10), grade II (11–20), grade III (21–40), and grade IV (>40). We defined high-burden PVS as grades III and IV. The inter-rater (kappa statistics: 0.86–0.90) and intra-rater (kappa: 0.90) reliability for PVS in the FHS is excellent.

MRI methods to determine white matter hyperintensity volume (WMHV), total cranial, and brain volume in the FHS have been previously described [14] using semiautomated modeling and segmentation procedures. The total cranial-to-brain volume ratio was used to account for head size differences, and white matter hyperintensity volume was expressed as a proportion of total cranial volume. Extensive WMH was defined as >1 age-specific standard deviation (SD) above the age-predicted value [14]. We used this threshold for extensive WMH burden considering previous work that related decreased cognitive function to WMHV 1 SD above the age-predicted value [17]. Inter-rater (0.90–0.94) and intra-rater reliability (0.98) for WMHV is excellent [14].

Covert brain infarcts (CBIs) were defined using the STRIVE consortium criteria, based on size (3–15 mm), location, imaging characteristics of the lesion, and being clinically asymptomatic [11]. The intra-rater and inter-rater reliability for CBI ratings in the FHS is excellent (kappa statistic: 0.73–0.90).

Cortical superficial siderosis (cSS) was defined as linear gyriform areas of low signal along the superficial layers of the cerebral cortex on blood-sensitive sequences (without the corresponding hyperintense signal on T1-weighted or fluid-attenuated inversion recovery image) that were noncontiguous with ICH [18].

### 2.5. Multi-Marker CSVD Score

Multi-marker CSVD score was calculated by adding the number of CSVD markers present in the MRI: CMBs, extensive WMH, high-burden PVS in BG or CSO, CBI, and cSS. The score ranged from 0 (no CSVD features) to 5 (all CSVD features present).

Due to the small number of participants with more than two markers, the multi-marker score was categorized as 0, 1, or 2+ markers for analysis.

### 2.6. Covariates

Age at the time of brain MRI acquisition, sex, body mass index (BMI), *APOE-ε4* allele presence, and prevalent diabetes defined as random blood glucose ≥ 200 mg/dl (11.1 mmol/L) or fasting blood sugar ≥ 126 mg/dL (7 mmol/L) or by the use of oral hypoglycemic agents or insulin for all cohorts. Hypertension, according to Seventh Report of the Joint National Committee on Prevention, Detection, Evaluation, and Treatment of High Blood Pressure Criteria, was defined as a systolic blood pressure of ≥140 mmHg, a diastolic blood pressure of ≥90 mmHg, or the use of antihypertensive medications [19]. Systolic and diastolic blood pressures were recorded as the mean of the FHS clinic physician’s two measurements. Readings were approximated to the nearest even number. Current smoking status was self-reported smoking of at least 1 cigarette per day within the year preceding examination. The use of preventive medications was self-reported.

### 2.7. Statistical Analysis

Continuous variables were presented as mean (SD) while categorical variables were presented as counts (percentages). The primary dependent measure was multi-marker CSVD score. We defined our exposures as follows: prevalent CVD or AF (participants who have prevalent CVD or prevalent AF), prevalent CVD (participants who have CVD independent of AF), prevalent AF (participants who have AF independent of CVD), and individual components of CVD (participants who have different components of CVD independent of AF). Multivariable logistic regression models with generalized logits were used to evaluate the relationship between prevalent CVD or AF, prevalent CVD, prevalent AF, and individual components of CVD with multi-marker CSVD score and individual CSVD markers. A score of zero (indicating no CSVD markers) was used as the reference group.

The primary model was adjusted for age at MRI, sex, FHS cohort, and time interval between MRI and clinic visit. The secondary model was additionally adjusted for hypertension, diabetes mellitus, BMI, cigarette smoking, statin use, and APOE ε4 allele. Subgroup analyses were performed to assess associations between prevalent CVD or AF with CSVD by age (<65 years vs. older), sex, hypertension status, and APOE ε4 allele presence.

Analyses were performed using SAS version 9.4 (SAS Institute, Cary, NC, USA). Two-sided *p* value < 0.05 was considered statistically significant. We applied a false discovery rate (FDR) correction using the q-value method to account for multiple testing [20]. A threshold of Q < 0.10, corresponding to an FDR of 10% was used to identify significant results.

## 3. Results

The participants were middle-aged and older adults (mean age: 59 ± 14 years, range: 26–96 years), with a balanced sex distribution (53.4% women). Three hundred and seventy-two (11%) participants had prevalent CVD or AF, 140 had prevalent AF (4%), 286 (8%) had prevalent CVD, 198 (6%) had prevalent CHD, and 34 (1%) had prevalent heart failure. Higher mean age, higher mean systolic blood pressure, presence of hypertension, statin use, prevalent CVD or AF, and CVD components were more likely in participants with higher multi-marker CSVD scores (Table 1). Supplementary Table S1 shows the clinical characteristics of included and excluded participants. The excluded participants were older, had higher prevalence of hypertension, and prevalent cardiovascular disease or atrial fibrillation.

### 3.1. Multivariable Regression Analyses (Table 2)

Participants with prevalent CVD or AF (OR: 1.38; CI: 1.05–1.84) and prevalent AF (OR: 1.86, CI: 1.24–2.80) had higher odds of having one CSVD marker in model 2 and after FDR adjustment compared to those with no CSVD markers Additionally, those with prevalent CVD, CHD, and heart failure had higher odds of having one CSVD marker and of having at least two CSVD markers, but associations were not significant.

### 3.2. Prevalent CVD and Individual CSVD Markers (Figure 2)

Significant associations were observed between CMBs and prevalent CVD or AF (OR: 1.82; 95% CI: 1.27, 2.63), prevalent AF (OR: 1.78; 95% CI: 1.10, 2.89), prevalent CVD (OR: 2.08; 95% CI: 1.41, 3.07), prevalent CHD (OR: 1.64; 95% CI: 1.05, 2.56), and prevalent heart failure (OR: 6.66; 95% CI: 3.19, 14) in model 2 and after FDR adjustment. Prevalent CVD, its components, and AF exhibited increased odds for CBI, though associations were only significant with prevalent CVD or AF (OR: 1.47; 95% CI: 1.02, 2.13) and prevalent AF (OR: 1.65; 95% CI: 1.02, 2.67). We could not perform analysis with cSS due to the small number.

### 3.3. Exploratory Analyses (Supplementary Table S2)

Exploratory analyses were limited by a smaller sample size in some subgroups, but we observed different associations. Prevalent CVD or AF was associated with higher odds for one CSVD marker in those who do not have hypertension compared to those who have hypertension, those not on treatment for hypertension compared to those receiving treatment, and in those who do not have APOE ε4 allele.

## 4. Discussion

In healthy community dwellers free of stroke and dementia, prevalent CVD or AF was associated with the presence of one CSVD marker. All the components of prevalent CVD and AF were associated with cerebral microbleeds, with the strongest association observed in participants who had prevalent heart failure. Exploratory analysis suggested that individuals with prevalent CVD or AF but without hypertension had stronger associations with CSVD burden.

As expected, individuals with a higher burden of CSVD were older and had proportion of vascular risk factors. The lower prevalence of atrial fibrillation in our study compared to previous estimates in the United States and worldwide [21,22] may be related to the younger age of the participants in our study (59 years versus 76.9 years [21]).

We observed the strongest association of all components of prevalent CVD and AF in participants with cerebral microbleeds. Although this relation may be related to the use of anticoagulants/antithrombotic medications, we were unable to explore this mechanism. Nonetheless, others have reported a similar association in patients with stroke [23]. In addition, although CMBs are considered hemorrhagic markers, they not only reflect an increased bleeding risk but also signal an increased risk of ischemic events [24].

Overall, findings from our study align with prior reports that observed associations between prevalent CVD, AF with silent brain infarcts, and CMBs [25,26]. We expand prior results by including a sample of individuals younger than those in previous reports (mean age: 59 years versus 76 years [25] and 75 years [26]), which highlights the increasing burden of subclinical vascular brain injury in younger people with cardiovascular disease.

Unlike earlier studies [7,27], we did not observe any association between prevalent CVD or AF with extensive WMHV, though effect size were stronger with heart failure.

The conducted exploratory analyses highlight the complexities in the relation of CVD and vascular brain injury. In a previous study [28], we observed that the *APOE-ε4* genotype was significantly associated with CVD, mirroring the finding of higher odds of ≥2 CSVD markers in participants with the *APOE-ε4* genotype in all components of CVD. In this study, the significant association between the *APOE-ε4*-negative genotype and one CSVD marker in participants with prevalent AF and prevalent CVD or AF is puzzling given prior findings [28,29,30] of association between the *APOE-ε4* genotype with CVD and AF and should be interpreted with caution, needing further exploration. This finding in participants who do not have hypertension may suggest that other risk factors may play a role in the association between CVD or AF with covert brain injury, or protective effect of medications for hypertension. Also, since we defined hypertension with a cut-off value of 140/90 mmHg, it is possible that lower blood pressure levels in people with CVD or AF is related to vascular brain injury and needs further exploration.

Noteworthy is the significant association between prevalent AF and one CSVD marker in those <65 years, which highlights that younger individuals with AF may have a higher burden of covert vascular brain injury, but these findings need replication.

Although prevalent heart failure consistently increased the odds for multi-marker CSVD score in all subgroups analysis, findings were limited by the small sample of individuals heart failure in the highest CSVD groups, thus limiting statistical power.

## 5. Limitations and Strengths of Our Study

The limitations of our study include its cross-sectional design, which hinders our ability to examine any causal relationship between prevalent CSVD or AF with multi-marker CSVD score. The predominant White constitution of our cohort limits generalizability to more diverse populations. The inability to assess the role of anticoagulant/antithrombotic medications in the associations between prevalent CVD or AF with CMBs, the small number of participants in our subgroup analysis, and multiple testing underscore the need for caution, as results should be viewed as hypothesis-generating and require replication in future studies. Further, it is possible that those with prevalent heart failure may have subclinical AF given that AF occurs commonly with heart failure.

The strengths of our study include our large sample size of community dwellers free of stroke and dementia and the reliable assessments of prevalent cardiovascular disease and AF and of markers of CSVD, with good-to-excellent reproducibility.

## 6. Conclusions

Prevalent CVD/AF are associated with subclinical CSVD, particularly with CMBs, in community-dwelling individuals free of stroke and dementia. In view of the strong relation of CSVD with dementia and stroke risk, our study brings attention to the need to recognize adverse brain health effects in patients with established CVD and AF, and of enhanced preventive efforts to minimize the risk of adverse clinical consequences.

## 7. Clinical Perspective

The relation of prevalent CVD and AF with vascular brain injury in subclinical stages represented by CSVD merits attention. CSVD is strongly associated with the risk of stroke and dementia. While patients with prevalent CVD and AF are likely to receive treatments for vascular risk factors, our findings bring attention to the need for the close monitoring of the treatment of vascular risk factors to ensure that treatment targets are met and sustained over time. The strongest association with prevalent heart failure may suggest that these patients are at an increased risk of subclinical vascular injury, which should spur further efforts and studies to protect brain health. Lastly, the results of exploratory analyses showing that the associations with CSVD were mainly observed in participants without hypertension need replication but may suggest that vascular risk factors other than hypertension play a role in subclinical vascular brain injury and need to be addressed.

## Figures and Tables

**Figure 2:**
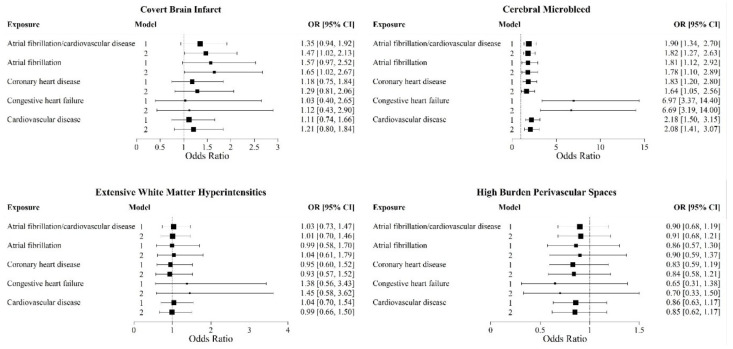
Multivariable logistic regression analysis of the association of prevalent CVD/AF with individual CSVD markers. Model 1 was adjusted for age at MRI, sex, FHS cohort, and time interval between MRI and clinic visit. Model 2 was additionally adjusted for hypertension, diabetes mellitus, BMI, cigarette smoking, statin use, and APO E ε4 allele presence. All analysis were adjusted for multiple testing using false discovery rate (FDR) correction.

**Table 1:** Sample characteristics of the study participants based on multi-marker CSVD score.

Clinical Characteristics	No CSVD Marker (N = 2336)	1 CSVD Marker (N = 773)	≥2 CSVD Markers (N = 304)
Male, n (%)	1089 (47)	366 (47)	134 (44)
Age at clinic exam, years, mean (SD)	53.3 (12.5)	61.3 (14.1)	69.8 (11)
Age at MRI, years, mean (SD)	55.4 (12.3)	63.2 (13.8)	71.4 (10.8)
FHS Cohort, n (%)			
Original	13 (1)	30 (4)	26 (9)
Offspring	761 (33)	399 (52)	223 (73)
Gen 3 and NOS	1491 (64)	308 (40)	54 (18)
Omni 1	71 (3)	36 (5)	7 (2)
Time between clinic exam and MRI, years, mean (SD)	1.6 (1.0)	1.4 (1.1)	1.2 (1.1)
Systolic blood pressure, mmHg, mean (SD)	120 (16)	124 (18)	132 (19)
Diastolic blood pressure, mmHg, mean (SD)	74 (10)	74(10)	72 (11)
Body mass index, kg/m^2^, mean (SD)	27.9 (5.4)	28.2 (5.5)	27.8 (5)
*APOE-ε4*+, n (%)	514 (22)	171 (22)	86 (28)
Smoking, n (%)	194 (8)	58 (8)	17 (6)
Hypertension, n (%)	721 (31)	377 (49)	200 (66)
Hypertension treatment, n (%)	573 (25)	321 (42)	161 (53)
Statin use, n (%)	577 (25)	277 (36)	118 (39)
Diabetes, n (%)	181 (8)	100 (13)	47 (15)
Prevalent cardiovascular disease or atrial fibrillation, n (%)	172 (7)	132 (17)	68 (22)
Prevalent cardiovascular disease, n (%)	128 (5)	104 (13)	54 (18)
Prevalent atrial fibrillation, n (%)	58 (2)	56 (7)	26 (9)
Prevalent coronary heart disease, n (%)	90 (4)	71 (9)	37 (12)
Prevalent heart failure, n (%)	10 (<1)	13 (2)	11 (4)

Abbreviations: FHS = Framingham Heart Study; MRI = magnetic resonance imaging; NOS = New Offspring Spouse; SD = standard deviation.

**Table 2:** Multivariable logistic regression analysis on the relationship between CVD and multi-marker CSVD score.

Predictor	Model ^a^	Multi-Maker CSVD Score		
		0 (N = 2336)	1 (N = 773) OR (95% CI)	≥2 (N = 304) OR (95% CI)
Prevalent cardiovascular disease or AF	1	1.00 (ref)	1.46 (1.12, 1.90) *^#^	1.19 (0.84, 1.69)
	2	1.00 (ref)	1.38 (1.05, 1.84) *^#^	1.25 (0.87, 1.81)
Prevalent AF	1	1.00 (ref)	1.78 (1.19, 2.66) *^#^	1.25 (0.74, 2.11)
	2	1.00 (ref)	1.86 (1.24, 2.80) *^#^	1.37 (0.80, 2.34)
Prevalent cardiovascular disease	1	1.00 (ref)	1.43 (1.06, 1.93) *^#^	1.18 (0.80, 1.73)
	2	1.00 (ref)	1.31 (0.96, 1.79)	1.22 (0.81, 1.83)
Prevalent coronary heart disease	1	1.00 (ref)	1.29 (0.91, 1.83)	1.06 (0.67, 1.66)
	2	1.00 (ref)	1.19 (0.83, 1.71)	1.09 (0.68, 1.73)
Prevalent heart failure	1	1.00 (ref)	1.76 (0.74, 4.21)	2.00 (0.78, 5.10)
	2	1.00 (ref)	1.77 (0.74, 4.23)	2.29 (0.85, 5.94)

CI = confidence interval; CSVD = cerebral small vessel disease; OR = odds ratio. ^a^ Model 1: adjusted for age at MRI, sex, FHS cohort, and time interval between MRI and clinic exam. Model 2: additionally adjusted for hypertension, smoking, diabetes, anti-lipid medication, BMI, and APOE ε4 allele. * *p* < 0.05, ^#^ q < 0.1.

## Data Availability

Requests to access de-identified data from the Framingham Heart Study can be made at https://www.framinghamheartstudy.org/fhs-for-researchers. The data used in this analyses was last assessed 26 June 2025.

## Supplementary Materials

The following supporting information can be downloaded at: https://www.mdpi.com/article/10.3390/brainsci15080813/s1, Table S1: Sample characteristics of included and excluded; Table S2: Subgroup analyses of risk factors on the association between prevalent CVD and atrial fibrillation with multi-marker CSVD score.
