# Supplementary material for: Prevalent Cardiovascular Disease and Atrial Fibrillation in Relation to Cerebral Small Vessel Disease Burden
Source: Brain Sci. 2025 Jul 29;15(8):813. doi: 10.3390/brainsci15080813 (PMC12385141; doi:10.3390/brainsci15080813)
Supplement: Supplementary file 1 [file brainsci-15-00813-s001.zip › brainsci-3757923-supplementary.pdf]

Supplementary Tables

Supplementary Table S1: Sample characteristics of included and excluded

**Clinical characteristics**	**Included (N=3413)**	**Excluded (N=297)**
Male, n (%)	1589 (47)	145 (49)
Age at clinic exam, years, mean (SD)	56.6 (13.8)	67.5 (14.6)
Age at MRI, years, mean (SD)	58.6 (13.6)	69.1 (14.2)
FHS Cohort, n (%)		
Original	69 (2)	49 (16)
Offspring	1383 (41)	156 (53)
Gen 3 & NOS	1847 (54.1)	64 (21.5)
OMNI 1	114 (3)	28(9)
Time between clinic exam & MRI, years, mean (SD)	1.5 (1)	1.1 (1.2)
Systolic blood pressure, mmHg, mean (SD)	122 (16)	130 (22)
Diastolic blood pressure, mmHg, mean (SD)	74 (10)	72 (10)
Body mass index, kg/m^2, mean (SD)	28.0 (5.4)	27.9 (5.1)
APOE-ε4+, n (%)	771(23)	70(24)
Smoking, n (%)	269 (8)	27 (9)
Hypertension, n (%)	1298 (38)	173 (58)
Hypertension treatment, n (%)	1055 (31)	150(51)
Statin use, n (%)	972 (28)	115 (39)
Diabetes, n (%)	328 (10)	39(13)
Cardiovascular disease or atrial fibrillation, n (%)	372 (11)	111 (37)
Cardiovascular disease, n (%)	286 (8)	98(33)
Atrial fibrillation, n (%)	140 (4)	33 (11)
Coronary heart disease, n (%)	198 (6)	37 (12)
Heart failure, n (%)	34 (1)	9 (3)
Cerebral microbleeds, n (%)	224 (6.6)	-
High burden PVS, n (%)	581 (17)	-
Extensive white matter hyperintensity, n (%)	394 (11.6)	-
Covert brain infarct, n (%)	248 (7.3)	-
Cortical superficial siderosis, n (%)	2 (0.06)	-

Supplementary Table S2: Subgroup analyses of risk factors on the association between prevalent CVD and atrial fibrillation with multi-marker CSVD score

Risk factor	1 marker	≥2 markers
	OR (95% CI)	OR (95% CI)
***APOE-ε4* positive n=771**<br><br>Prevalent CVD or AF	1.13 (0.63, 2.01)	1.57 (0.82, 3.02)

Risk factor	1 marker	≥2 markers
	OR (95% CI)	OR (95% CI)
Prevalent AF	1.67 (0.70, 4.00)	1.39 (0.48, 4.00)
Prevalent CHD	1.05 (0.52, 2.15)	1.29 (0.59, 2.84)
Prevalent Heart Failure	1.81 (0.15, 21.58)	2.52 (0.24, 26.68)
Prevalent CVD	0.92 (0.48, 1.78)	1.43 (0.71, 2.88)
***APOE-ε4* negative n=2518**		
Prevalent CVD or AF	1.52 (1.12, 2.07) [*#]	1.12 (0.72, 1.72)
Prevalent AF	1.94 (1.22, 3.09) [*#]	1.39 (0.75, 2.57)
Prevalent CHD	1.31 (0.87, 1.97)	0.94 (0.53, 1.65)
Prevalent Heart Failure	1.77 (0.7, 4.49)	2.04 (0.71, 5.80)
Prevalent CVD	1.53 (1.08, 2.16) [*]	1.08 (0.67, 1.74)
**Age < 65 years[c] n=2298**		
Prevalent CVD or AF	1.57 (0.97, 2.53)	1.61 (0.65, 3.96)
Prevalent AF	2.4 (1.16, 4.96) [*#]	1.82 (0.4, 8.15)
Prevalent CHD	1.07 (0.51, 2.22)	0.85 (0.19, 3.75)
Prevalent CVD	1.27 (0.7, 2.29)	1.33 (0.45, 3.95)
**Age ≥ 65 years n=1115**		
Prevalent CVD or AF	1.32 (0.96, 1.83)	1.1 (0.75, 1.62)
Prevalent AF	1.44 (0.9, 2.32)	1.1 (0.63, 1.94)
Prevalent CHD	1.31 (0.87, 1.97)	1.08 (0.67, 1.76)
Prevalent Heart Failure	1.41 (0.57, 3.46)	1.81 (0.71, 4.62)
Prevalent CVD	1.43 (1.01, 2.04)	1.16 (0.76, 1.77)
**Presence of Hypertension n=1298**<br>Prevalent CVD or AF	1.11 (0.8, 1.54)	0.84 (0.55, 1.28)
Prevalent AF	1.41 (0.86, 2.31)	0.88 (0.47, 1.67)
Prevalent CHD	1.03 (0.69, 1.55)	0.76 (0.45, 1.28)
Prevalent Heart Failure	1.43 (0.48, 4.26)	1.64 (0.52, 5.23)
Prevalent CVD	1.15 (0.80, 1.64)	0.89 (0.57, 1.4)
**No Hypertension n=2115**<br>Prevalent CVD or AF	2.08 (1.31, 3.31) [*#]	2.11 (1.09, 4.07) [*#]
Prevalent AF	2.35 (1.19, 4.64) [*#]	2.45 (0.98, 6.09)
Prevalent CHD	1.92 (0.94, 3.94)	2.05 (0.83, 5.11)
Prevalent Heart Failure	2.42 (0.58, 10.1)	2.6 (0.52, 13.01)
Prevalent CVD	1.97 (1.13, 3.45) [*]	1.82 (0.84, 3.93)
**Treatment for Hypertension, n=1055**<br>Prevalent CVD or AF	1.13 (0.79, 1.61)	1.04 (0.67, 1.62)
Prevalent AF	1.43 (0.84, 2.43)	1.12 (0.58, 2.16)
Prevalent CHD	1.08 (0.71, 1.65)	0.91 (0.53, 1.56)
Prevalent Heart Failure	1.47 (0.45, 4.81)	2.16 (0.64, 7.31)
Prevalent CVD	1.21 (0.83, 1.77)	1.08 (0.67, 1.74)

Risk factor	1 marker	≥2 markers
	OR (95% CI)	OR (95% CI)
**No treatment for hypertension, n=2357**		
Prevalent CVD or AF	1.80 (1.18, 2.75) *#	1.34 (0.73, 2.46)
Prevalent AF	2.14 (1.16, 3.97)	1.37 (0.57, 3.32)
Prevalent CHD	1.48 (0.79, 2.79)	1.29 (0.56, 2.95)
Prevalent Heart Failure	2.12 (0.59, 7.64)	1.64 (0.35, 7.56)
Prevalent CVD	1.60 (0.97, 2.64)	1.24 (0.62, 2.49)
**Male, n=1589**		
Prevalent CVD or AF	1.40 (0.99, 2.00)	1.11 (0.68, 1.82)
Prevalent AF	1.77 (1.07, 2.92) *#	1.29 (0.65, 2.56)
Prevalent CHD	1.25 (0.80, 1.94)	1.06 (0.59, 1.91)
Prevalent Heart Failure	0.91 (0.25, 3.29)	1.72 (0.49, 6.09)
Prevalent CVD	1.33 (0.90, 1.98)	0.89 (0.51, 1.55)
**Female, n=1824**		
Prevalent CVD or AF	1.51 (1.00, 2.28)	1.34 (0.8, 2.23)
Prevalent AF	1.79 (0.92, 3.47)	1.23 (0.54, 2.81)
Prevalent CHD	1.35 (0.76, 2.39)	1.07 (0.52, 2.18)
Prevalent Heart Failure	3.04 (0.84, 11.08)	2.86 (0.69, 11.89)
Prevalent CVD	1.58 (1.00, 2.49)	1.63 (0.94, 2.85)

Abbreviations: CI = confidence interval; CSVD = cerebral small vessel disease; OR = odds ratio. 0 CSVD marker was used as reference. Results are adjusted for age at MRI, sex, Framingham Heart Study (FHS) cohort and time interval between MRI and clinic exam.

*P<0.05 #q<0.1

[c] Prevalent heart failure and CSVD score not done in participants < 65 years because very few numbers of participants < 65 years had heart failure. CVD: cardiovascular disease, AF: Atrial fibrillation.
